# Discovery colleges in youth mental health systems: developmentally adapted recovery-oriented learning environments

**DOI:** 10.1186/s13033-026-00718-9

**Published:** 2026-06-23

**Authors:** Jared Omundo, Manuel Föcker, Simon Alexander Stiehl, Shiblu Miah, Michael Schulz, Orkan Okan

**Affiliations:** 1 TUM School of Medicine and Health Department of Health and Sport Sciences, Technical University of Munich, Munich, Germany; 2 Recovery College Gütersloh (RC GT-OWL), LWL-Klinikums, Gütersloh Haus 55, Buxelstraße 50, 33334 Gütersloh, Germany; 3 LWL Klinikum Gütersloh, Pflegerische Abteilungsleitung Allgemeinpsychiatrie, Honorar-Professur an der Fachhochschule der Diakonie, Gütersloh, Bielefeld, Germany; 4 Department of Child and Adolescent Psychiatry, University Hospital Münster, Münster, Germany; 5 LWL-University Hospital Hamm for Child and Adolescent Psychiatry, Campus Gütersloh, Ruhr-University Bochum, Gütersloh, Germany; 6 Medizinische Fakultät, Medizinische Hochschule Brandenburg Theodor Fontane, Fehrbelliner Straße 38, 16816 Neuruppin, Germany; 7 Hochschule Osnabrück – University of Applied Science, Albrechtstraße 30, 49076 Osnabrück, Germany; 8 Recovery College Osnabrück (RCO), KAFF e.V, Am Speicher 3a, 49090 Osnabrück Osnabrück, Germany; 9 Recovery and Discovery Colleges (NHS) RECOIL – Flourish in Education, Luton, UK; 10 Technical University of Munich, School of Medicine and Health, Department of Health and Sport Sciences, WHO Collaborating Centre for Health Literacy, Munich, Germany

**Keywords:** Discovery Colleges, Youth mental health, Recovery-oriented practice, Recovery Colleges, Co production, Mental health literacy, Youth mental health systems, Service integration

## Abstract

**Background:**

Youth mental health systems worldwide face increasing demand, workforce shortages, and fragmented pathways to care. Addressing these challenges requires approaches that extend beyond specialist clinical treatment to incorporate prevention, participation, education, and recovery-oriented support. While Recovery Colleges are increasingly established within adult mental health services, their youth-adapted counterparts commonly referred to as Discovery Colleges remain underexplored within youth mental health systems research. This narrative review synthesises emerging literature to examine the conceptual foundations, developmental adaptations, and potential system-level role of Discovery Colleges within youth mental health care.

**Methods:**

A narrative review approach was employed to synthesise emerging literature on Discovery Colleges and related recovery-oriented educational initiatives. Peer-reviewed publications, programme descriptions, and implementation reports were analysed to examine the defining principles, developmental relevance, organisational characteristics, and system-level implications of Discovery Colleges within youth mental health contexts.

**Results:**

The synthesis identified four interrelated conceptual domains characterising Discovery Colleges: (1) recovery-oriented learning models and developmental adaptation; (2) mechanisms of engagement and participation; (3) organisational and professional implications; and (4) cross-sector positioning within youth mental health ecosystems. The findings suggest that Discovery Colleges can be conceptualised as developmentally responsive and recovery-oriented learning environments operating at the intersection of education, community support, and mental health care. The review further highlights important tensions relating to implementation, safeguarding, fidelity, sustainability, and cross-sector integration.

**Conclusions:**

Discovery Colleges may represent a promising recovery-oriented innovation within youth mental health systems by reframing mental health support as a collaborative, participatory, and educational process rather than solely a clinical intervention. However, the current evidence base remains limited and largely exploratory. Further empirical, developmental, and implementation-focused research is required to evaluate their effectiveness, sustainability, and long-term contribution to integrated youth mental health systems.

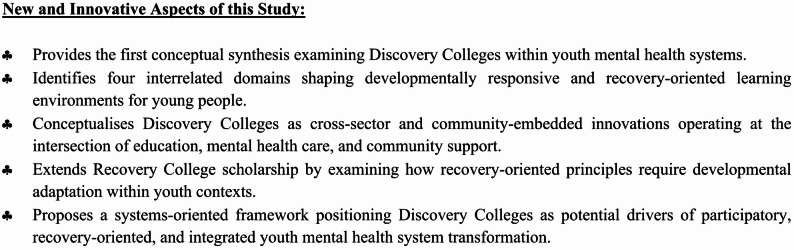

## Introduction

The mental health of children, adolescents, and young adults represents a major global public health challenge [40, 55]. For the purposes of this review, youth is understood broadly as individuals navigating adolescence and young adulthood, approximately between the ages of 11 and 25 years. These developmental periods are characterised by substantial social, cognitive, emotional, and identity-related change, while simultaneously representing critical phases for the emergence of mental health difficulties. In recent years, increasing social pressures, academic demands, and persistent barriers to timely mental health support have exposed the limitations of predominantly treatment-oriented and reactive models of care, which remain insufficiently equipped to address escalating and early emerging mental health needs among young people [11, 58].

Within this broader context, the COVID-19 pandemic further amplified pre-existing structural and psychosocial vulnerabilities within youth mental health systems. Longitudinal evidence, including findings from the COPSY study, demonstrated substantial declines in health-related quality of life alongside increases in emotional and behavioural difficulties, particularly among socioeconomically disadvantaged groups [45–47]. Increased loneliness and disruptions to peer relationships further weakened developmental resources associated with belonging, autonomy, and identity formation during adolescence. Simultaneously, interruptions to mental health service provision, delayed care, and reduced accessibility highlighted ongoing structural limitations within youth mental health systems [10, 36]. Despite growing recognition of the importance of prevention and early intervention, substantial barriers to accessing mental health support persist globally and within Germany, including long waiting times, limited specialist capacity, and ongoing stigma surrounding help-seeking [18, 26, 61]. Many mental health systems remain fragmented, under-resourced, and predominantly reactive, prioritising symptom management over prevention, participation, relational engagement, and recovery-oriented care [58]. Accessibility gaps are particularly pronounced in rural and underserved regions, where service shortages and long travel distances disrupt continuity of care [53]. These systemic limitations underscore the need for more accessible, participatory, and community-based approaches capable of complementing traditional clinical services and supporting engagement at earlier stages of distress [40].

Within this context, recovery-oriented approaches have increasingly gained attention as alternatives to predominantly biomedical and deficit-focused models of mental health care. Rather than conceptualising mental health solely in terms of symptom reduction, recovery-oriented practice emphasises mental health as a personal, relational, and socially embedded process through which individuals pursue meaningful and self-directed lives despite ongoing challenges [1, 50]. Central principles include empowerment, co-production, participation, peer support, and recognition of lived experience as a legitimate form of expertise [14, 29]. Within adult mental health services, Recovery Colleges and Empowerment Colleges (RECs) have emerged as prominent educational implementations of these principles, reframing mental health support as a collaborative learning process rather than solely a clinical intervention [29, 51, 52]. Grounded in co-production, peer involvement, and adult learning approaches, Recovery Colleges provide non-clinical educational environments in which people with lived experience, professionals, and community members learn together on more equal terms. Emerging implementation and fidelity frameworks have further strengthened the conceptualisation of Recovery Colleges as recovery-oriented organisational innovations rather than isolated educational programmes [22, 34]. In particular, the RECOLLECT framework identifies core fidelity components including co-production, strengths-based learning, peer involvement, educational orientation, social connectedness, and community integration [57]. Similarly, the Implementing Recovery through Organisational Change (ImROC) programme positions Recovery Colleges as mechanisms of broader organisational and cultural transformation within mental health systems [34, 42]. Evidence from adult mental health services suggests that Recovery Colleges may foster empowerment, connectedness, self-efficacy, and engagement while simultaneously challenging traditional hierarchies and professional power structures within mental health care [13, 35, 38, 57]. More recently, youth-adapted versions of these models, commonly referred to as Discovery Colleges (DCs), have emerged in countries including Australia, Ireland, and the United Kingdom [16, 59, 60].

**Fig. 1:**
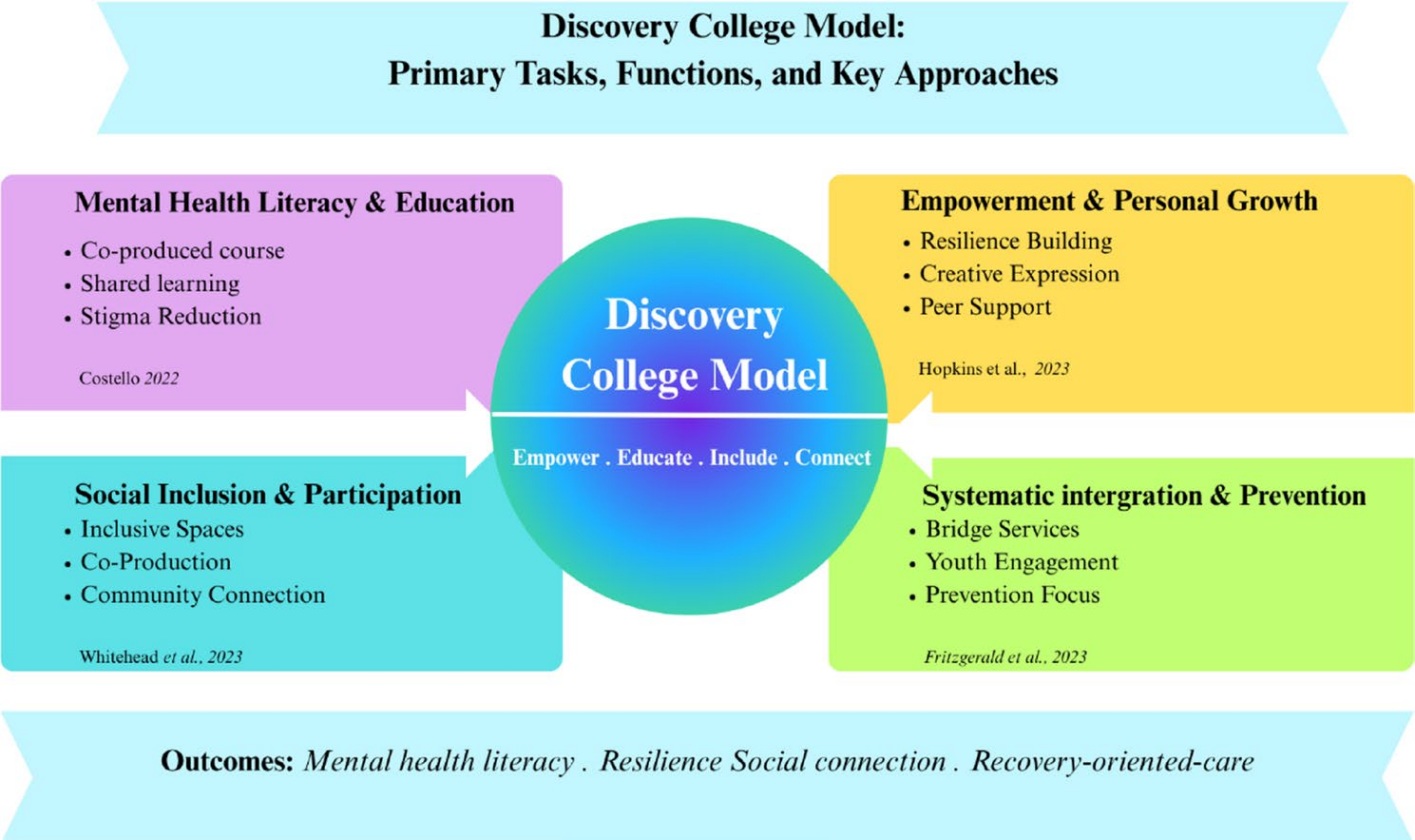
Core Functions, Mechanisms, and Recovery-Oriented Principles of Discovery Colleges (Authors own illustration)

Discovery Colleges (DCs) are recovery-oriented educational initiatives designed for children, adolescents, and young adults that combine mental health literacy, peer learning, and co-produced learning environments within non-clinical settings [59]. Rather than positioning young people as passive recipients of treatment, Discovery Colleges conceptualise participants as active learners who collaboratively engage with topics relating to mental health, wellbeing, coping, and recovery. Courses are typically developed and delivered through collaboration between professionals and individuals with lived experience, reflecting principles of co-production, participatory learning, and recovery-oriented practice [16, 59]. As illustrated in Fig. 1, Discovery Colleges can be conceptualised as recovery-oriented learning environments organised around several interconnected functions, including mental health literacy and education, empowerment and personal growth, social inclusion and participation, and system integration and prevention. These domains reflect the broader recovery-oriented principles underpinning Discovery Colleges, including co-production, lived experience involvement, inclusivity, and non-clinical learning environments. Beyond individual-level outcomes, the model further positions Discovery Colleges as relational and community-embedded infrastructures capable of strengthening engagement, social connection, and recovery-oriented pathways across youth mental health systems.

Despite their conceptual alignment with the broader Recovery College movement, the implementation of Discovery Colleges within youth mental health systems introduces important developmental and organisational considerations. Youth contexts are characterised by identity formation, educational participation, evolving autonomy, family involvement, and complex peer dynamics, all of which may influence how recovery-oriented educational approaches are experienced and implemented [32, 59], . However, Discovery Colleges remain comparatively underexplored within youth mental health research, particularly in Germany [52]. Existing literature remains fragmented, with limited theoretical discussion regarding developmental adaptation, implementation processes, mechanisms of engagement, and system-level implications [25, 30]. As a result, important questions remain regarding how recovery-oriented educational models can be meaningfully translated into youth mental health systems while maintaining participatory and non-pathologising principles.

### Aim

This article presents a conceptual narrative synthesis of Discovery Colleges within youth mental health systems. Specifically, the review aims to:

1. Situate Discovery Colleges within recovery-oriented and youth mental health frameworks.
2. Examine their defining principles, developmental adaptations, and mechanisms of engagement.
3. Explore their implications for organisational practice, service delivery, and youth mental health system transformation.

By synthesising emerging literature and conceptual perspectives, the review contributes to ongoing discussions regarding how youth mental health systems may evolve beyond predominantly treatment-oriented approaches toward more participatory, relational, and recovery-oriented models of support.

## Methodology

This study adopted a conceptual narrative review approach to examine Discovery Colleges (DCs) as an emerging recovery-oriented innovation within youth mental health systems. A narrative and interpretive review methodology was considered particularly appropriate given the interdisciplinary, heterogeneous, and still evolving nature of the field, which currently consists of diverse empirical, theoretical, and practice-based literature [44, 54]. Relevant literature was identified through targeted searches of major academic databases, including PubMed, Scopus, and Google Scholar, alongside examination of reference lists from key publications in the field. The review focused primarily on literature published between 2018 and 2025, reflecting the period during which youth-oriented adaptations of Recovery College models have received increasing scholarly and practical attention. Searches were guided by terms related to the central concepts of the review, including “Discovery College”, “Recovery College”, “youth mental health”, “co-production”, “peer learning”, “mental health literacy”, and “recovery-oriented practice”.

Given the limited evidence base relating specifically to Discovery Colleges, the review incorporated both youth-focused Discovery College literature and broader Recovery College research from adult mental health contexts. Including adult Recovery College literature enabled examination of how established recovery-oriented principles, implementation frameworks, and fidelity criteria may be transferable to youth mental health settings, while also identifying areas requiring developmental adaptation. In addition to peer-reviewed academic literature, organisational reports, programme descriptions, implementation documents, and service evaluations were consulted to capture emerging developments not yet fully represented within the scientific literature. Rather than applying rigid inclusion and exclusion criteria, literature was selected based on its relevance to the aims of the review. Particular attention was given to publications addressing recovery-oriented principles, co-production, peer learning, implementation processes, organisational change, developmental adaptation, and system-level implications of Discovery Colleges and related educational mental health initiatives. The analysis was conducted through iterative reading, interpretive comparison, and narrative synthesis of sources to explore relationships, tensions, and recurring themes across diverse forms of evidence [44]. Particular emphasis was placed on examining how Discovery Colleges are positioned in relation to broader Recovery College frameworks and how recovery-oriented principles may require reinterpretation within youth mental health contexts. Through this interpretive process, recurring patterns were identified and organised into overarching domains relating to recovery-oriented learning models, mechanisms of engagement, organisational culture, and system integration. These domains subsequently informed the analytical structure of the review.

## Results

**Fig. 2:**
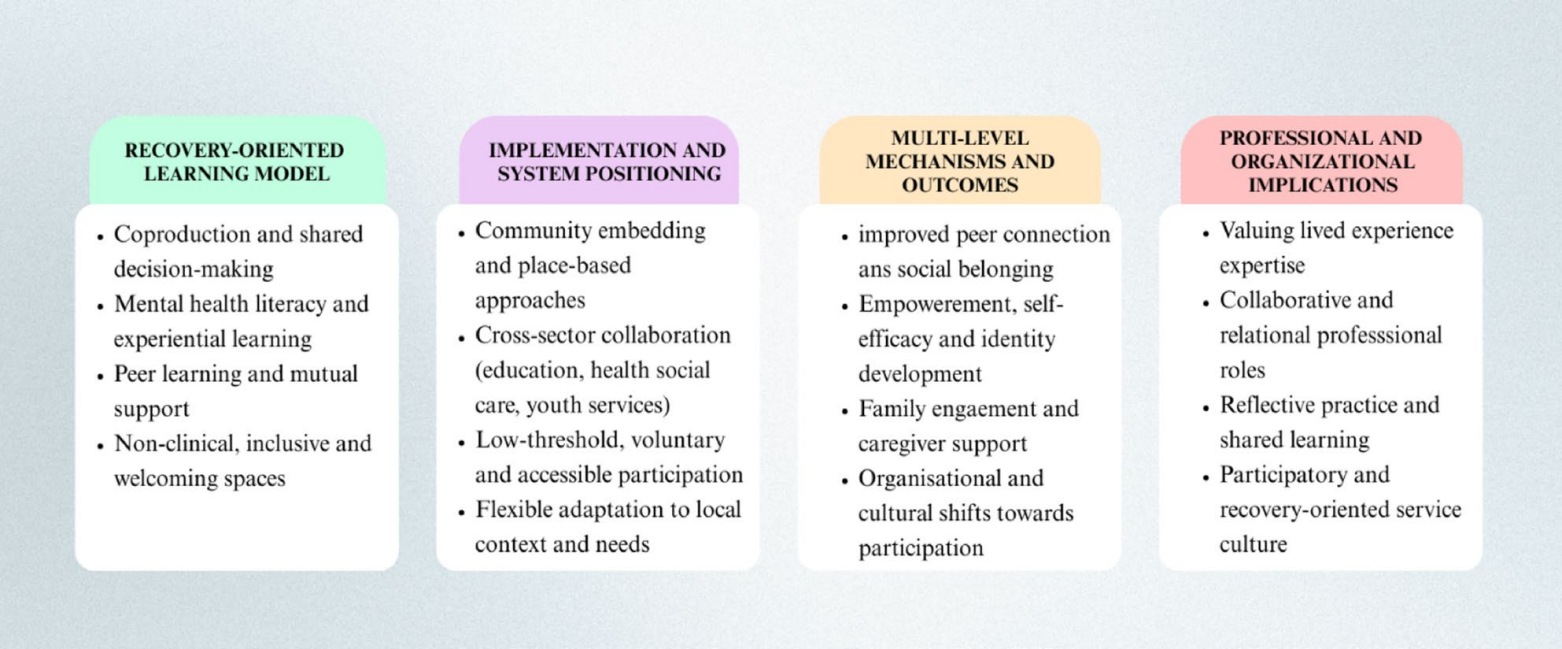
Key Themes Emerging from the Narrative Synthesis of Discovery Colleges in Youth Mental Health Systems (Authors own illustration)

The body of literature examining Discovery Colleges (DCs) within youth mental health systems remains relatively limited but is gradually expanding across several international contexts. Existing publications primarily reflect the early implementation and developmental stage of youth-adapted Recovery College models and consist largely of conceptual papers, programme descriptions, qualitative evaluations, implementation reports, and practice-based accounts. Although the current evidence base remains methodologically heterogeneous, the literature provides important insights into how recovery-oriented educational approaches are being adapted and positioned within youth mental health systems. The literature included in this review comprised a limited number of publications directly examining Discovery Colleges or youth-oriented Recovery College initiatives, alongside a broader body of Recovery College research derived from adult mental health services. The inclusion of adult Recovery College literature was important for examining how established recovery-oriented principles, implementation frameworks, and pedagogical assumptions may translate into youth mental health contexts and where developmental adaptation may be required. Across the reviewed literature, Discovery Colleges were consistently positioned not simply as youth versions of adult Recovery Colleges, but as recovery-oriented learning environments situated within broader developmental, educational, relational, and community contexts. Interpretive analysis of the literature identified four interrelated conceptual domains through which Discovery Colleges are currently understood and positioned within youth mental health systems: (1) recovery-oriented ethos and learning model; (2) implementation and system positioning; (3) multi-level mechanisms and outcomes; and (4) professional and organisational implications (see Fig. 2)

A small but significant group of publications provided substantive insights into the design, implementation, and experiential dimensions of Discovery Colleges, while also highlighting ongoing questions relating to participation, co-production, peer learning, organisational integration, and youth engagement. The relatively small number of youth-specific studies reflects the emerging nature of Discovery Colleges as a service innovation rather than restrictive inclusion criteria. Much of the available evidence remains exploratory and practice-based, with limited longitudinal, comparative, or outcome-focused research currently available. Consequently, the literature presently offers stronger insight into implementation processes, experiential perspectives, and recovery-oriented principles than into long-term effectiveness or system-level outcomes. The publications informing this synthesis (*n* = 9) are summarised in Table 1.

**Table 1:** Key Publications Informing the Conceptual Synthesis of Discovery Colleges

Author	Year	Context	Type of Publication	Contribution to Conceptual Understanding
Beckers & Koopmans	2025	Netherlands	Qualitative evaluation	Demonstrated how Recovery Colleges foster empowerment, participatory learning, and inclusive recovery-oriented environments
Bellemare et al.	2024	Canada	Qualitative interviews	Highlighted organisational and professional shifts associated with collaborative and relational recovery-oriented practice
Fitzgerald et al.	2023	Ireland	Narrative review	Synthesised emerging Discovery College models and identified their relevance for youth mental health literacy, empowerment, and participatory engagement
Whitehead et al.	2023	UK	Observational study	Showed that young participants value peer learning, emotional validation, relational safety, and shared experiential learning environments
Hopkins et al.	2023	Australia	Implementation case study	Described the integration of Discovery Colleges within youth mental health services and highlighted cross-sector and non-clinical positioning
Costello	2021	Ireland	Descriptive case study	Presented the early development of a youth Discovery College emphasising co-production, accessibility, and collaborative learning
Whitley et al.	2019	Canada	Qualitative study	Conceptualised Recovery Colleges as spaces supporting identity development, connectedness, and social inclusion
Hopkins et al.	2018	Australia	Programme evaluation	Reported improvements in empowerment, participation, and engagement associated with Recovery College participation
Crowther et al.	2018	UK	Mixed-methods evaluation	Demonstrated recovery-oriented and organisational impacts of Recovery Colleges, including changes in service culture and relational practice

### Recovery-oriented ethos and learning model

Across the literature, Discovery Colleges (DCs) are conceptualised as developmentally adapted recovery-oriented educational models informed by the broader Recovery College framework [12, 16, 24]. Recovery Colleges emerged within adult mental health services as non-clinical learning environments grounded in co-production, strengths-based approaches, peer involvement, and collaborative knowledge exchange [34, 42]. Rather than positioning individuals primarily as patients or service users, these models conceptualise participants as active learners engaged in educational processes relating to mental health, wellbeing, and recovery [34, 57]. Emerging implementation and fidelity frameworks have further contributed to the understanding of Recovery Colleges as organisational and cultural innovations within mental health systems [22, 27, 57]. In particular, the RECOLLECT framework identifies key fidelity components including co-production, strengths-based learning, peer involvement, social connectedness, community orientation, equality, and educational rather than clinical positioning [22]. Similarly, the Implementing Recovery through Organisational Change (ImROC) programme positions Recovery Colleges as mechanisms of broader organisational transformation capable of challenging traditional professional hierarchies and promoting participatory service cultures ([42]; Perkins et al., 2018). Adult Recovery College research included in this review further describes outcomes relating to empowerment, identity development, connectedness, and social participation [4, 13].

**Fig. 3:**
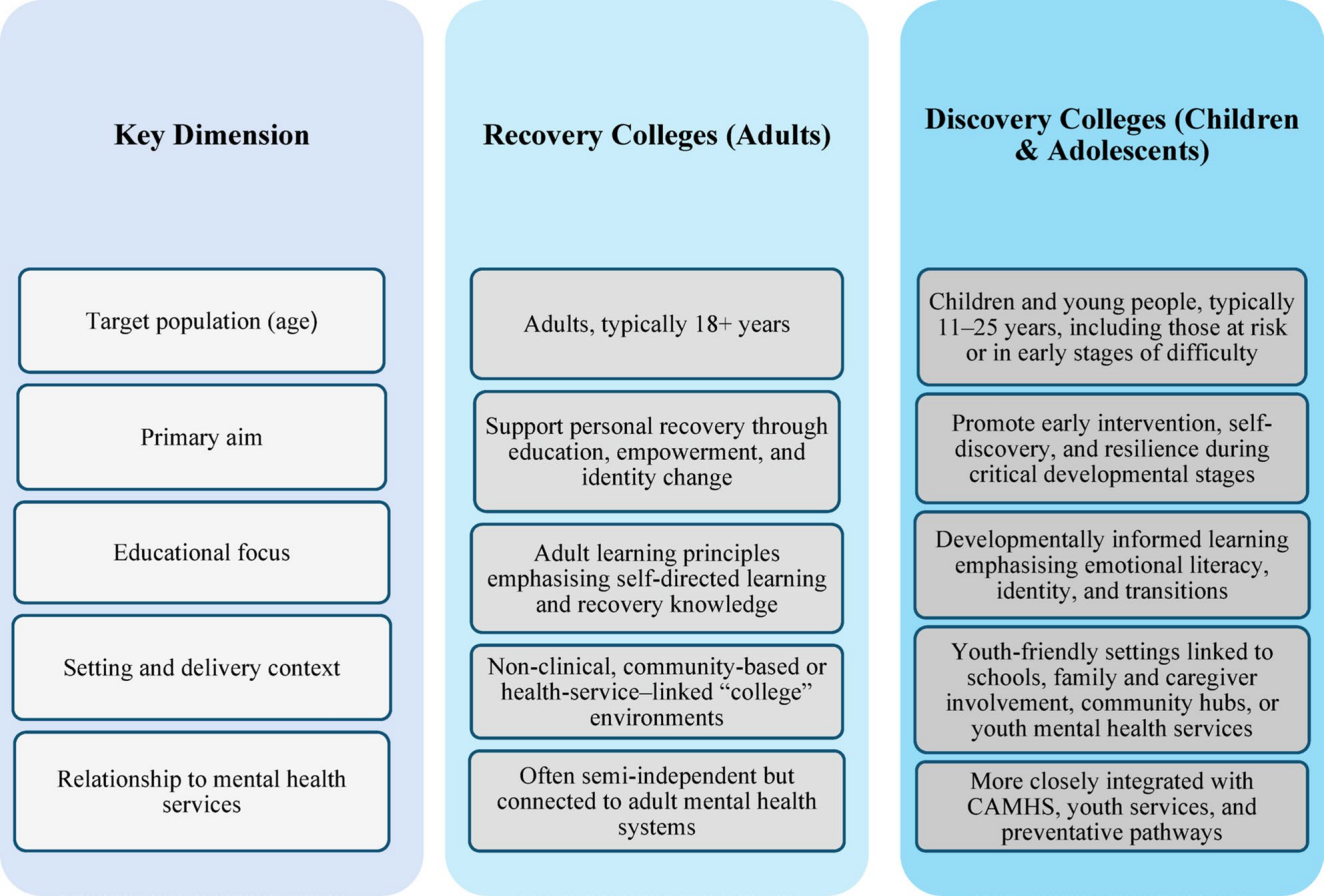
Comparative Developmental and Organisational Dimensions of Recovery Colleges and Discovery Colleges (Omundo et al., in peer review)

Within youth mental health contexts, Discovery Colleges appear to retain many of these recovery-oriented principles while adapting them to developmental, educational, and relational contexts specific to children, adolescents, and young adults. Youth-focused publications describe programme structures incorporating co-production, participatory learning, peer engagement, and strengths-based approaches tailored to younger populations. Costello [12], for example, describes the first Irish Discovery College as structured around collaborative course development involving young people, families, and professionals. Similarly, Hopkins et al., [25] report that Australian youth Discovery Colleges were embedded within existing youth mental health services while maintaining a non-clinical identity centred on accessibility, participation, and relational engagement. Several publications further emphasise the importance of mental health literacy, emotional expression, peer learning, and collaborative learning environments within Discovery College models [16, 59]. Young participants reportedly valued opportunities for connection, emotional openness, and engagement within less hierarchical and less pathologising environments [59]. As illustrated in Fig. 3, Discovery Colleges appear to retain several foundational principles associated with adult Recovery Colleges, including co-production, peer learning, empowerment, and non-clinical educational engagement. However, the reviewed literature also highlights important developmental and organisational distinctions relating to educational participation, family involvement, safeguarding, developmental transitions, and relationships with youth mental health services.

### Multi-level mechanisms and outcomes

No large-scale controlled trials specifically examining youth-focused Discovery Colleges were identified within the scope of this review, and rigorous longitudinal or comparative evaluations remain limited [24, 59]. Nevertheless, the available literature consistently describes a range of psychosocial, relational, and service-related outcomes associated with participation in Discovery College programmes [59]. Existing findings suggest that Discovery Colleges may operate through interrelated mechanisms involving participation, peer connection, collaborative learning, empowerment, and non-clinical engagement [22, 34, 57]. At the individual level, young people frequently report increased confidence, improved coping strategies, enhanced emotional expression, stronger peer connection, and greater mental health literacy following participation in Discovery College activities [12, 16, 59]. Several publications further suggest that participatory and peer-based learning environments may reduce feelings of isolation while strengthening relational belonging and engagement [13, 57]. Importantly, these outcomes appear to emerge not solely through the delivery of mental health information, but through collaborative and relational learning processes involving peers, professionals, and lived experience perspectives within less hierarchical environments [34]. Such mechanisms may hold particular relevance during adolescence and young adulthood, developmental periods characterised by identity formation, autonomy development, and heightened importance of peer relationships [31–33, 48].

At the organisational level, findings suggest that Discovery Colleges may also influence how youth mental health services are experienced and delivered. Hopkins et al., [24], for example, describe positive experiences among both young people and staff, particularly in relation to accessibility, flexibility, and non-clinical engagement. The literature further suggests that Discovery Colleges may function as low-threshold and relationally accessible entry points into mental health support, potentially facilitating earlier engagement among young people who may otherwise avoid formal clinical services due to stigma or distrust of traditional care systems [32, 59]. Evidence from adult Recovery College research included within this review similarly documents outcomes relating to empowerment, hope, identity development, social connectedness, and improved engagement with services [4, 13, 60].

### Implementation and positioning within systems

Existing literature suggests that Discovery Colleges (DCs) are consistently conceptualised as recovery-oriented learning environments operating at the intersection of mental health care, education, and community support systems [12, 16, 25]. While implementation models vary across contexts, Discovery Colleges are generally described as community-based or service-affiliated initiatives that intentionally maintain a non-clinical and low-threshold identity [59]. This positioning enables voluntary participation without requiring formal diagnosis or referral pathways and may reduce barriers associated with traditional mental health services, including stigma and limited accessibility [32]. Several publications emphasise the importance of embedding Discovery Colleges within broader youth mental health ecosystems while preserving their educational and participatory orientation. Hopkins et al., [24], for example, describe Australian Discovery Colleges as integrated within existing youth mental health services as part of broader service transformation initiatives, while still functioning as relational and accessible learning environments. Similarly, Costello [12], highlights the importance of cross-sector collaboration involving young people, families, professionals, educational institutions, and community organisations in the implementation of the first Irish Discovery College.

The literature further suggests that Discovery Colleges may occupy an intermediary position between prevention, early intervention, community engagement, and recovery-oriented support [24]. Their placement within familiar and less formal environments may facilitate earlier engagement among young people who might otherwise avoid specialist services or experience difficulties accessing conventional care pathways [59]. In this sense, Discovery Colleges appear to function not only as educational interventions but also as relational access points embedded within broader youth support systems [24, 59]. At the same time, publications consistently describe variability in governance structures, funding arrangements, implementation processes, and degrees of system integration across Discovery College models [16]. Costello [12], for example, identifies challenges relating to cross-sector coordination, sustaining co-production processes, and securing long-term institutional support. Similar tensions are reflected within adult Recovery College literature, where programmes embedded within existing service systems must simultaneously preserve non-clinical, participatory, and educational identities [4, 13]. Importantly, the literature suggests that implementation within youth contexts may involve additional developmental and organisational complexities [32, 59].

### Professional roles and organisational culture

The reviewed literature further suggests that Discovery Colleges (DCs) and related Recovery College models are consistently described as collaborative learning environments involving partnerships between professionals, individuals with lived experience, young people, families, and community members [24, 59, 60]. Central to these approaches is the principle of co-production, in which course development, facilitation, and educational engagement are undertaken collaboratively rather than through traditional top-down service structures [24, 57]. Youth-focused publications particularly emphasise participatory approaches involving young people, families, and professionals in course design, delivery, and evaluation [12, 16, 24]. Within Discovery College settings, professional roles are conceptualised less in terms of expert-led clinical intervention and more as facilitative, relational, and collaborative forms of engagement [24]. Rather than occupying exclusively authoritative positions, professionals participate within shared learning environments that recognise lived experience and experiential knowledge as valuable forms of expertise. Young participants similarly described Discovery College environments as less hierarchical, less clinical, and more relational compared with traditional mental health service settings [59]. The literature further highlights the importance of peer learning and lived experience involvement within Discovery College models [24, 59]. Peer-based learning environments appear to support emotional openness, shared understanding, and relational connectedness among participants [34, 57]. However, publications also suggest that peer involvement within youth settings may require additional developmental and organisational considerations, including safeguarding responsibilities, age-appropriate facilitation, and support structures tailored to adolescents and young adults [24, 41, 48]. Evidence from adult Recovery College research included within this review similarly documents broader organisational and cultural changes associated with programme implementation. Crowther et al., (2018), for example, report increased recovery-oriented practices and more collaborative service cultures within host organisations. Beckers and Koopmans [4], further describe organisational environments characterised by stronger emphasis on empowerment, participation, and shared decision-making following Recovery College implementation. Similarly, Bellemare et al., [6] found that professionals involved in Recovery College programmes described shifts in attitudes toward lived experience expertise, collaboration, and professional identity.

## Discussion

The review indicates that Discovery Colleges retain several core principles associated with adult Recovery Colleges, including co-production, peer learning, strengths-based approaches, and non-clinical educational engagement. However, the findings also suggest that the implementation of these principles within youth contexts involves important developmental, relational, and organisational adaptations. In particular, issues relating to identity formation, educational participation, autonomy, peer dynamics, and family involvement may shape how recovery-oriented learning environments are experienced and operationalised among younger populations. At the same time, Discovery Colleges appear to occupy a distinct intermediary position between prevention, early intervention, community engagement, and recovery-oriented support. Their non-clinical and participatory positioning may provide alternative pathways into mental health support for young people who experience barriers to accessing traditional services. The following sections discuss these findings in relation to developmental adaptation, mechanisms of engagement, ecosystem positioning, organisational implications, and future directions for research and implementation.

### Discovery colleges as developmentally adapted recovery-oriented models

The literature suggests that Discovery Colleges (DCs) represent developmentally adapted extensions of the Recovery College model within youth mental health contexts. While adult Recovery Colleges have been associated with empowerment, identity reconstruction, social connectedness, and recovery-oriented participations [13, 38], youth-oriented models appear to incorporate additional attention to developmental processes characteristic of adolescence and young adulthood [32, 41]. These include identity formation, educational transitions, peer relationships, family involvement, and evolving autonomy during periods of substantial social and psychological change [48]. Central to Discovery Colleges is the reframing of participants as active learners rather than passive recipients of care [59]. This orientation challenges deficit-focused and exclusively treatment-oriented understandings of mental health support by emphasising participation, agency, co-production, and collaborative learning [12, 25]. In this sense, Discovery Colleges align closely with recovery-oriented frameworks such as CHIME, which conceptualise recovery in relation to connectedness, hope, identity, meaning, and empowerment [29]. These principles are reflected not only in course content but also in broader pedagogical approaches centred on peer learning, experiential knowledge, and collaborative participation [7, 59].

At the same time, the findings suggest that several assumptions underpinning adult Recovery Colleges may require reconsideration within youth contexts. For example, while adult Recovery Colleges often emphasise a transition from “patient” to “student” identity, many adolescents and young adults already occupy educational roles, potentially altering the meaning and mechanisms of empowerment associated with participation. Similarly, principles such as self-directed learning and peer facilitation may require additional developmental support, safeguarding structures, and age-appropriate relational approaches within youth-oriented settings. Several core principles associated with adult Recovery Colleges therefore appear to require developmental reinterpretation within youth-oriented Discovery College models. As summarised in Table 2, these adaptations reflect differences in autonomy, educational participation, identity development, peer relationships, and safeguarding responsibilities across youth mental health contexts.

**Table 2:** Developmental Adaptations of Recovery College Principles Within Discovery College Models

Shared Recovery College Principles	Developmental Adaptations within Discovery Colleges
Co-production	Safeguarding and developmentally supported participation
Peer learning	Age-appropriate and relationally supported facilitation
Educational orientation	Educational transitions and school-related dynamics
Empowerment	Evolving autonomy and supported decision-making
Recovery-oriented identity	Ongoing identity formation and self-development

Educational participation may also represent a more complex and potentially ambivalent domain within youth contexts than within adult Recovery Colleges. Although Discovery Colleges adopt educational frameworks intended to reduce clinical stigma and promote engagement, many young people already experience substantial pressures within formal educational systems. Experiences of academic stress, school disengagement, school avoidance, or exclusion may therefore shape how educationally framed mental health initiatives are perceived and experienced. Consequently, the extent to which Discovery Colleges function as empowering alternatives to institutional environments, or risk reproducing educational hierarchies and performance expectations, remains an important area for further conceptual and empirical exploration. Discovery Colleges should therefore not simply be understood as youth versions of adult Recovery Colleges, but rather as developmentally responsive reinterpretations of recovery-oriented educational practice situated within broader youth mental health ecosystems.

### Peer support and co-production within youth contexts

Peer support and co-production emerged across the reviewed literature as central principles underpinning Discovery Colleges (DCs) and broader Recovery College approaches. Within recovery-oriented frameworks, these principles are understood as mechanisms for reducing hierarchical distinctions, strengthening lived experience involvement, and promoting collaborative learning and participation [29]. The findings of this review suggest that these principles remain highly relevant within youth mental health contexts, although their implementation may require important developmental adaptation. Youth-focused Discovery College models frequently described co-production as involving collaborative participation between young people, professionals, families, and community stakeholders in course design, delivery, and evaluation [12, 25]. Similarly, peer learning environments were associated with opportunities for emotional openness, shared understanding, relational belonging, and reduced isolation among participants [59]. Such collaborative environments may provide less hierarchical and less pathologising forms of engagement than traditional clinical settings, particularly during adolescence and young adulthood when peer relationships, identity formation, and autonomy development hold heightened developmental importance.

At the same time, the implementation of peer support and co-production within youth settings introduces additional developmental, relational, and organisational complexities. While peer facilitation and lived experience involvement constitute central fidelity principles within adult Recovery Colleges, their application within youth contexts may require age-appropriate facilitation models, safeguarding structures, and developmentally supported participation. Adolescents and young adults differ considerably in terms of emotional maturity, autonomy, and vulnerability, potentially shaping how peer relationships and collaborative participation are experienced within Discovery College environments. Furthermore, co-production within youth mental health systems may involve ongoing negotiation between participatory ideals and adult responsibilities. Although Discovery Colleges seek to reduce hierarchical distinctions and promote shared expertise, professionals and organisations continue to hold safeguarding and ethical responsibilities toward younger participants. Future research should therefore examine how peer facilitation, lived experience involvement, and collaborative participation can be operationalised in developmentally appropriate and ethically sustainable ways within youth-oriented recovery frameworks.

### Mechanisms of engagement and participation

The findings of this review suggest that Discovery Colleges (DCs) operate through mechanisms extending beyond individual educational outcomes and may influence relational, organisational, and participatory processes within youth mental health systems. Rather than functioning solely as psychoeducational interventions, Discovery Colleges appear to create collaborative learning environments in which engagement emerges through peer connection, participation, relational safety, and shared meaning-making. At the individual level, participation in Discovery College activities has been associated with increased confidence, improved coping strategies, enhanced mental health literacy, and stronger peer connection [12, 59]. Young people further appear to value opportunities for emotional openness, experiential learning, and participation within less hierarchical environments. These findings align with recovery-oriented educational approaches emphasising collaborative knowledge exchange, lived experience involvement, and strengths-based learning. Such mechanisms may hold particular relevance during adolescence and young adulthood, developmental periods characterised by identity formation, evolving autonomy, and heightened importance of peer relationships. The review further suggests that Discovery Colleges may operate as low-threshold and relationally accessible forms of support. Their non-clinical and participatory orientation may reduce barriers associated with stigma, fear of pathologisation, or distrust of formal mental health services, thereby facilitating earlier engagement among young people who might otherwise avoid traditional systems of care [59]. These findings align with broader Youth Mental Health youth mental health literature emphasising the importance of accessibility, relational trust, and non-stigmatising engagement environments in promoting help-seeking and sustained participation [2, 23, 31, 32].

At the organisational level, Recovery College research further indicates that such programmes may influence professional attitudes, service cultures, and collaborative practices. Existing evidence suggests that engagement with Recovery College models can strengthen recognition of lived experience expertise, encourage more collaborative relationships between professionals and participants, and support recovery-oriented approaches within services [6, 9, 13, 57]. Within youth mental health systems, these relational and organisational shifts may contribute to movement away from hierarchical service models toward more participatory and developmentally responsive forms of care. Cumulatively, the findings suggest that Discovery Colleges may function not only as educational programmes, but also as relational infrastructures capable of influencing individual experiences, social participation, and organisational cultures within youth mental health systems. However, the absence of longitudinal, comparative, and controlled evaluations specific to youth Discovery Colleges currently limits conclusions regarding their long-term effectiveness, mechanisms of impact, and broader system-level implications.

### Discovery colleges within youth mental health ecosystems

**Fig. 4:**
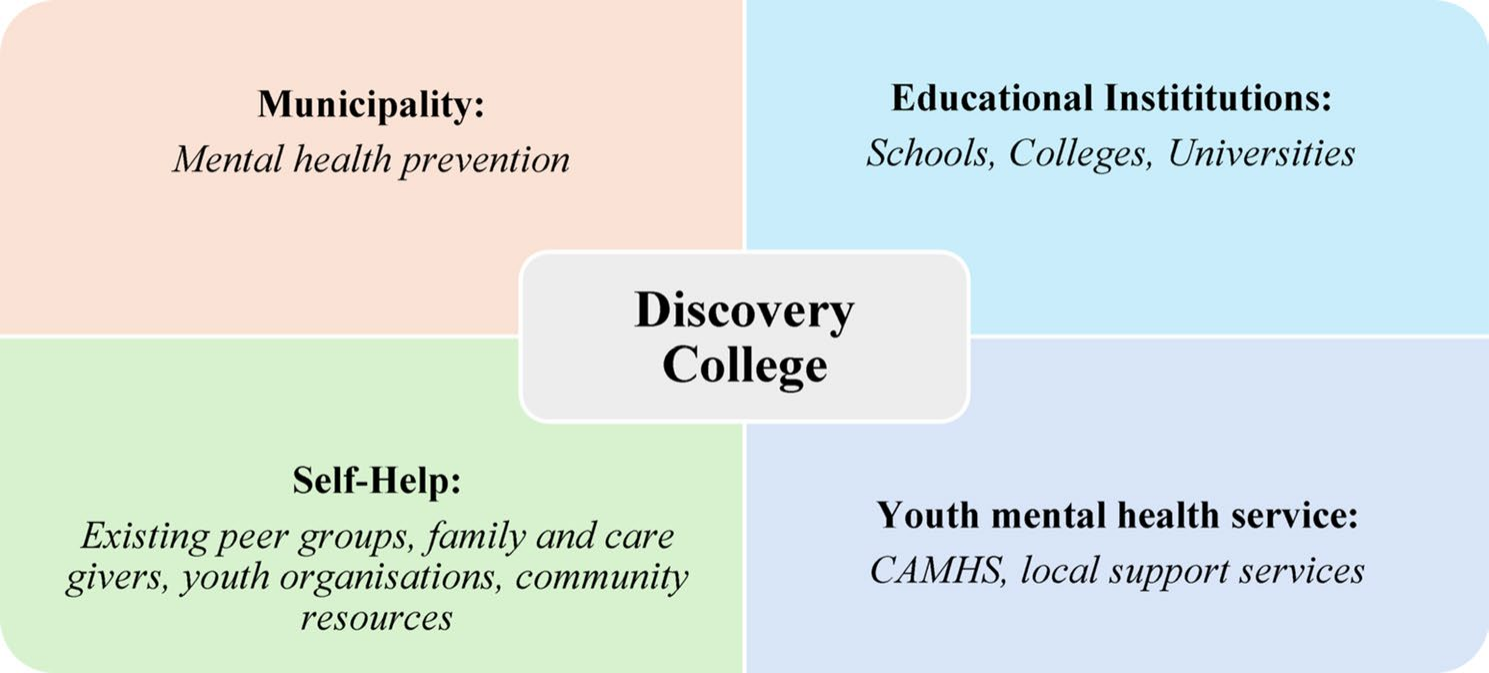
Discovery Colleges as Community-Embedded and Cross-Sector Learning Environments within Youth Mental Health Ecosystems (Authors’ own illustration)

Another key insight emerging from this synthesis concerns the positioning of Discovery Colleges (DCs) within broader youth mental health ecosystems. Across the reviewed literature, Discovery Colleges are consistently situated at the intersection of education, community support, and specialist mental health services. Figure 4 conceptualises Discovery Colleges as cross-sector and community-embedded learning environments operating at the interface between education, youth mental health services, public health structures, and community-based support systems. This intermediary positioning allows them to function as accessible, non-clinical, and community-oriented learning environments while remaining connected to existing youth mental health infrastructures. In this sense, Discovery Colleges may operate not only as educational initiatives, but also as relational access points embedded within wider systems of youth support and early intervention.

Their positioning aligns closely with contemporary youth mental health frameworks emphasising prevention, early intervention, accessibility, and developmentally appropriate community-based responses [8, 32]. The voluntary and non-clinical nature of Discovery Colleges may reduce barriers associated with stigma, fear of pathologisation, and restrictive referral thresholds, thereby facilitating engagement among young people who experience distress or functional difficulties but may not access specialist services through conventional pathways [17, 28]. In this regard, Discovery Colleges may complement stepped-care approaches prioritising accessible and lower-intensity forms of support alongside escalation pathways when clinically indicated [37, 49].

Importantly, the findings further suggest that Discovery Colleges operate within developmental ecosystems shaped not only by mental health services, but also by schools, families, peer networks, and community structures [48]. Unlike adult Recovery Colleges, youth-oriented models are embedded within contexts where educational participation, family involvement, safeguarding responsibilities, and social belonging may substantially influence engagement processes [24, 59]. Schools may therefore represent both important access pathways and potential sites of tension for Discovery College implementation. While school-based partnerships may improve accessibility and early engagement, they may also risk reproducing institutional hierarchies, performance pressures, or exclusionary dynamics experienced by some young people within educational settings. These tensions highlight the importance of maintaining relational, participatory, and non-pathologising environments when integrating Discovery Colleges within educational systems. At the same time, this cross-sector positioning may introduce important organisational and implementation challenges. Initiatives operating between education, health, and community systems frequently encounter ambiguity relating to governance structures, commissioning responsibilities, funding arrangements, and institutional accountability [19, 56]. Without clearer policy integration and sustainable funding models, Discovery Colleges may risk remaining peripheral or locally dependent initiatives reliant on individual champions and short-term project funding [22]. Tensions may also emerge between maintaining participatory and non-clinical identities and increasing institutionalisation within formal service structures. In synthesis, these findings suggest that Discovery Colleges may represent attempts to bridge gaps between youth mental health care, education, and community participation through more relational, accessible, and developmentally responsive forms of support. However, embedding such approaches sustainably within youth mental health systems will likely require greater conceptual clarity, cross-sector collaboration, and structural investment at organisational and policy levels.

### Organisational and professional implications

A further dimension highlighted by this synthesis concerns the transformation of professional roles and organisational cultures associated with Discovery College (DC) implementation. A defining feature of Discovery Colleges is the redistribution of expertise through co-production, peer learning, and lived experience involvement. Within these settings, individuals with lived experience contribute not only as participants, but also as educators, facilitators, and collaborators in programme development, thereby challenging traditional hierarchies between professional and experiential knowledge [7, 39]. The findings suggest that peer facilitation and lived experience involvement may represent important mechanisms of engagement within Discovery Colleges. Peer educators may contribute to relational openness, model hope and self-efficacy, and reduce perceived hierarchies between participants and professionals [15]. Young people participating in Discovery College environments frequently described valuing collaborative, relatable, and less pathologising forms of interaction, particularly opportunities for shared learning and emotional expression with peers and facilitators [59].

At the same time, the review indicates that peer involvement within youth contexts may require important developmental and organisational adaptations [24, 59]. Unlike adult Recovery Colleges, youth-oriented models operate within settings shaped by safeguarding responsibilities, varying developmental stages, evolving autonomy, and ongoing family involvement [48]. Questions relating to who facilitates courses, how lived experience expertise is operationalised, and how peer relationships are supported therefore become particularly important within youth settings. Age-appropriate facilitation structures, relational support mechanisms, and safeguarding frameworks may be necessary to ensure that peer learning environments remain both participatory and developmentally responsive. The findings further suggest that several assumptions underpinning adult Recovery College models may require reconsideration within youth mental health systems [24, 59]. For example, while adult Recovery Colleges often emphasise transitions from “patient” to “student” identities, many young people already occupy educational roles and institutional learning environments. Similarly, assumptions relating to self-directed learning and peer facilitation may operate differently within adolescent populations characterised by varying developmental autonomy and school-related experiences. Key examples of how recovery-oriented principles may require developmental reinterpretation within youth contexts are summarised in Table 3.

**Table 3:** Developmental Adaptations of Recovery College Principles within Discovery Colleges

Adult Recovery College Assumption	Youth-Specific Developmental Consideration
Patient-to-student identity shift	Many young people already occupy educational identities
Self-directed learning	Developmental autonomy and decision-making capacities vary
Peer facilitation	Safeguarding and age-appropriate peer support structures required
Educational participation	School-related stress, fatigue, and school avoidance may influence engagement
Individual recovery orientation	Family systems and caregiver involvement remain highly influential

For professionals, participation in Discovery Colleges may require a shift from expert-driven practice toward more facilitative, collaborative, and partnership-oriented roles. Such shifts align with broader recovery-oriented workforce developments emphasising relational accountability, co-production, and participatory care [6, 9]. For psychiatric nurses, youth mental health practitioners, educators, and community workers, Discovery Colleges may therefore offer opportunities to engage in more community-facing and recovery-oriented forms of practice [20, 21]. At the organisational level, sustaining these collaborative approaches may depend on supportive institutional conditions, including training structures, recognition of peer roles, leadership support, and integration within broader service systems. Without such support, the participatory ethos underpinning Discovery Colleges may become difficult to maintain within traditionally hierarchical and clinically oriented service environments. Collectively, these findings suggest that Discovery Colleges may function not only as educational interventions for young people, but also as potential catalysts for broader cultural and organisational transformation within youth mental health systems.

## Strengths and limitations

To our knowledge, this review represents the first conceptual synthesis examining Discovery Colleges (DCs) from a youth mental health systems perspective. A key strength lies in its integrative and systems-oriented approach, which enabled the inclusion of empirical studies, programme descriptions, implementation reports, and practice-based literature. This allowed the review to conceptualise Discovery Colleges as recovery-oriented, community-embedded, and developmentally responsive innovations operating across educational, public health, community, and mental health service contexts. The synthesis further contributes to emerging discussions on participatory and recovery-oriented youth mental health care by highlighting the roles of co-production, peer learning, mental health literacy, and non-clinical engagement.

Several limitations and conceptual challenges should also be considered. First, the evidence base relating specifically to youth-oriented Discovery Colleges remains limited and methodologically heterogeneous. Existing studies are predominantly qualitative, descriptive, or cross-sectional, with few focusing exclusively on adolescent populations. Consequently, much of the current conceptual grounding continues to rely on adult Recovery College literature [13, 59], which may not fully capture developmental processes unique to youth contexts. Second, important questions remain regarding the transferability of adult Recovery College principles into youth mental health systems. Assumptions relating to self-directed participation, peer facilitation, educational identity shifts, and individual recovery processes may operate differently within developmental contexts shaped by evolving autonomy, safeguarding responsibilities, family involvement, and educational dependency. Educationally framed interventions may also risk reproducing institutional pressures or exclusionary dynamics, particularly for young people experiencing school-related stress, disengagement, or school avoidance. School- or service-based implementation may therefore unintentionally recreate hierarchies that Discovery Colleges seek to challenge. Third, although co-production and lived experience involvement are central principles within Discovery Colleges, their implementation may remain challenging within institutional systems. Participatory approaches risk becoming tokenistic if young people’s contributions are not meaningfully integrated into decision-making and organisational processes.

Similarly, balancing authentic participation with safeguarding and professional responsibilities may represent an ongoing tension within youth-oriented recovery models. Fourth, variability in implementation contexts, governance structures, funding arrangements, and degrees of system integration limits comparability across studies [22, 56]. Questions regarding fidelity and adaptation also remain unresolved, particularly concerning how far Recovery College principles can be developmentally adapted without altering core recovery-oriented characteristics. Finally, the available evidence primarily reports short-term and self-reported outcomes. Recovery-oriented constructs such as empowerment, hope, connectedness, participation, and identity development may not be adequately captured through traditional symptom-focused evaluation frameworks [3]. In addition, as a narrative synthesis, this review did not apply formal systematic review procedures such as risk-of-bias assessment. The conclusions should therefore be interpreted as conceptually informed rather than definitively evaluative.

## Implications for research and youth mental health systems

Despite the limited evidence base, this synthesis highlights several important implications for future research and youth mental health system development. Existing research on Discovery Colleges (DCs) remains largely exploratory, qualitative, and cross-sectional, limiting understanding of long-term outcomes, mechanisms of impact, and broader system-level effects. Future studies should therefore prioritise longitudinal, comparative, and mixed-methods designs capable of examining recovery-oriented, developmental, and relational outcomes across diverse youth populations and service contexts. Future evaluation frameworks should move beyond symptom-focused measures to include indicators more closely aligned with the participatory ethos of Discovery Colleges, including empowerment, identity development, peer belonging, social participation, self-efficacy, and navigation of support systems [57]. Greater attention should also be given to organisational and system-level processes, including co-production practices, accessibility, service culture, and cross-sector collaboration. The findings further highlight the need for greater conceptual and empirical clarity regarding the developmental adaptation of Recovery College principles within youth contexts. Core principles such as peer learning, co-production, and educational participation may operate differently within settings shaped by evolving autonomy, safeguarding responsibilities, family involvement, and educational dependency. Future research should therefore examine how peer facilitation, lived experience involvement, and collaborative participation can be implemented in developmentally appropriate and ethically sustainable ways.

Additional research is also needed to better understand implementation processes across organisational and cultural contexts. Questions relating to governance, sustainable funding, workforce development, implementation fidelity, and cross-sector integration remain largely unresolved [22, 27]. Without clearer policy alignment and institutional support, Discovery Colleges risk remaining peripheral or locally dependent initiatives rather than sustainably embedded components of youth mental health systems. At the same time, the low-threshold, non-clinical, and participatory orientation of Discovery Colleges positions them as potentially valuable complements to stepped-care and integrated care approaches, particularly in contexts characterised by rising demand, limited accessibility, and workforce pressures [22, 56]. However, realising this potential will likely require stable funding structures, organisational investment, and sustained cross-sector collaboration. Equity considerations also remain critical, as young people experiencing socioeconomic disadvantage or structural barriers may require targeted outreach and additional support to ensure equitable participation [5, 43]. As summarised in Table 4, several interconnected strategic challenges remain central to the future development of Discovery Colleges, including evidence generation, developmental adaptation, implementation fidelity, system integration, and sustainability. Addressing these domains will be essential if Discovery Colleges are to move beyond locally driven innovations toward more systematically embedded components of youth mental health systems.

**Table 4:** Strategic Challenges and Future Directions for Discovery Colleges within Youth Mental Health Systems. (Source: Authors own work)

Strategic Domain	Key Challenges	System Implications	Future Directions
Evidence & Evaluation	Limited longitudinal, comparative, and youth-specific evidence; reliance on symptom-focused outcomes	Incomplete understanding of long-term and system-level impact	Develop recovery-oriented, developmental, and theory-informed evaluation frameworks
Implementation & Model Integrity	Variability in delivery models and developmental adaptation	Reduced comparability, scalability, and implementation consistency	Define core recovery-oriented principles alongside context-sensitive adaptations
System Integration & Governance	Fragmented commissioning, policy alignment, and cross-sector coordination	Risk of peripheral or short-term implementation	Establish integrated governance, commissioning, and cross-sector collaboration models
Sustainability & Funding	Reliance on short-term or project-based funding	Threats to continuity, workforce development, and long-term embedding	Develop sustainable funding pathways and policy-level institutional support

## Conclusion

Discovery Colleges (DCs) represent an emerging attempt to extend recovery-oriented principles into youth mental health systems through developmentally responsive, participatory, and community-based learning environments. Drawing on principles such as co-production, peer learning, experiential knowledge, and non-clinical engagement, Discovery Colleges appear to offer alternatives to predominantly treatment-focused models of care. The findings of this review suggest that Discovery Colleges should not simply be understood as youth versions of adult Recovery Colleges, but rather as developmentally adapted reinterpretations of recovery-oriented educational practice situated within broader youth mental health ecosystems. Their implementation within contexts shaped by identity formation, educational participation, family involvement, safeguarding responsibilities, and evolving autonomy introduces opportunities and challenges distinct from adult Recovery College models. Positioned at the intersection of education, community support, and mental health care, Discovery Colleges may support contemporary youth mental health reform efforts emphasising accessibility, prevention, participation, and cross-sector collaboration. At the same time, important questions remain regarding implementation fidelity, governance, sustainability, safeguarding, and long-term effectiveness. The current evidence base remains limited and largely exploratory, highlighting the need for further conceptual, empirical, and implementation-focused research. In summary, Discovery Colleges represent a promising but still evolving innovation within youth mental health care. If supported through rigorous evaluation, sustainable funding, and broader policy integration, they may contribute to more accessible, participatory, and developmentally responsive youth mental health systems.

## Data Availability

No datasets were generated or analysed during the current study.
